# Psychophysiological Responses to Stress after Stress Management Training in Patients with Rheumatoid Arthritis

**DOI:** 10.1371/journal.pone.0027432

**Published:** 2011-12-06

**Authors:** Sabine J. M. de Brouwer, Floris W. Kraaimaat, Fred C. G. J. Sweep, Rogier T. Donders, Agnes Eijsbouts, Saskia van Koulil, Piet L. C. M. van Riel, Andrea W. M. Evers

**Affiliations:** 1 Department of Medical Psychology, Radboud University Nijmegen Medical Centre, Nijmegen, The Netherlands; 2 Department of Laboratory Medicine, Radboud University Nijmegen Medical Centre, Nijmegen, The Netherlands; 3 Department of Epidemiology, Biostatistics and HTA, Radboud University Nijmegen Medical Centre, Nijmegen, The Netherlands; 4 Department of Rheumatology, Sint Maartenskliniek, Nijmegen, The Netherlands; 5 Department of Rheumatology, Radboud University Nijmegen Medical Centre, Nijmegen, The Netherlands; Federal University of Rio de Janeiro, Brazil

## Abstract

**Background:**

Stress management interventions may prove useful in preventing the detrimental effects of stress on health. This study assessed the effects of a stress management intervention on the psychophysiological response to stress in patients with rheumatoid arthritis (RA).

**Methods:**

Seventy-four patients with RA, who were randomly assigned to either a control group or a group that received short-term stress management training, performed a standardized psychosocial stress task (Trier Social Stress Test; TSST) 1 week after the stress management training and at a 9-week follow-up. Psychological and physical functioning, and the acute psychophysiological response to the stress test were assessed.

**Results:**

Patients in the intervention group showed significantly lower psychological distress levels of anxiety after the training than did the controls. While there were no between-group differences in stress-induced tension levels, and autonomic (α-amylase) or endocrine (cortisol) responses to the stress test 1 week after the intervention, levels of stress-induced tension and cortisol were significantly lower in the intervention group at the 9-week follow-up. Overall, the response to the intervention was particularly evident in a subgroup of patients with a psychological risk profile.

**Conclusion:**

A relatively short stress management intervention can improve psychological functioning and influences the psychophysiological response to stress in patients with RA, particularly those psychologically at risk. These findings might help understand how stress can affect health and the role of individual differences in stress responsiveness.

**Trial Registration:**

TrialRegister.nl NTR1193

## Introduction

The aetiology of rheumatoid arthritis (RA), a chronic inflammatory systemic disease that affects 1% of the general population [1], [2], remains poorly understood. Despite the growing spectrum of pharmacological therapies aimed at reducing disease activity [3], many patients continue to suffer from pain, fatigue, functional disability, and an overall poor quality of life [4]. One of the factors believed to play a role in the initiation, maintenance, and exacerbation of RA is psychological stress [5], [6]. Evidence is accumulating that stress-evoked physiological changes, brought about by activation of the two main branches of the stress response system, the autonomic nervous system (ANS) and the hypothalamus-pituitary-adrenal (HPA) axis, might have detrimental effects on disease activity and health [7]–[10]. This has led to growing interest into the effects of stress management interventions on physiological outcomes. Stress-reducing psychological interventions aimed at modifying stress appraisal and decreasing subjective anxiety might alter autonomic arousal (e.g., decrease heart rate and galvanic responses, and increase tonic vasodilation) and influence neuroendocrine activity (e.g., lower cortisol levels) [11]–[14]. Alleviating the physiological response to a stressor could be particularly relevant in clinical populations, specifically in patients with immune-mediated diseases, such as RA. Although evidence is limited, there are indications that stress management interventions might affect basal autonomic or endocrine parameters, such as norepinephrine levels, urinary free cortisol output, serum dehydroepiandrosterone sulphate, or testosterone levels in patients with HIV and cancer [15]–[21].

Psychological interventions, such as multimodal cognitive-behavioral therapy (CBT), biofeedback, stress management training, or emotional disclosure, have generally led to modest improvements in psychological and physical functioning in patients with RA, with similar effects for the different types of interventions [4], [22]–[25]. Only incidental effects have been found on biological measures of disease, such as C-reactive protein (CRP) and erythrocyte sedimentation rate (ESR) [26]–[28]. Medical and methodological explanations have been searched for this lack of uniform effects of psychological interventions on biological measures, such as disease status, medication regimen, and used time frame to assess physiological stress measures. However, there is also relatively consistent support that inter-individual variation in psychological risk factors also play a role [29], [30]. Specifically, previous research increasingly indicates the importance of evaluating psychological risk factors when investigating treatment outcome, such as the experience of interpersonal stress and levels of depression [29], [31]. For instance, there is increasing evidence that patients at risk, for example those who report being sensitive to stress or who have heightened levels of distress (e.g., heightened anxiety and depression), are especially prone to the detrimental effects of stress on disease activity and accompanying physical symptoms [32], [33]. Moreover, stress-induced changes in physiological function are particularly observed in these groups of patients psychologically at risk [29], [31], [34]. Although there is preliminary evidence that stress management interventions can influence the acute psychophysiological response to stress in healthy individuals [35], [36], it is not known whether such interventions alter the acute-phase psychophysiological response to a stressful event in immune-comprised patients with chronic inflammatory diseases, such as RA.

In this study, we examined the effects of a short-term individual stress management intervention on the self-reported, sympathetic, and neuroendocrine response to a validated psychosocial stress test (Trier Social Stress Task, TSST) in patients with RA and in a subsample of patients at risk of heightened anxiety and depression. We hypothesized that patients in the intervention group, particularly those at risk, would show reduced levels of distress and a diminished psychophysiological response to acute psychosocial stress compared with controls both after the intervention and at the 9-week follow-up after prolonged use of the stress management techniques.

## Materials and Methods

### Ethics statement

The protocol for this trial and supporting CONSORT checklist are available as supporting information; see Checklist S1 and Protocol S1. The study protocol was approved by the regional medical ethics committee (CMO Regio Arnhem-Nijmegen) and registered in The Netherlands National Trial Register (NTR 1193). Written informed consent was obtained from all participants.

### Participants

Patients with RA were recruited from the Department of Rheumatology at the Radboud University Nijmegen Medical Centre and the St Maartenskliniek in Nijmegen, the Netherlands. Inclusion criteria were a diagnosis of RA according to the American Rheumatism Association 1987 classification criteria [37] and a minimum age of 18. Exclusion criteria were severe physical comorbidity (e.g., major cardiac problems, psoriasis, malignancies, severe respiratory or renal insufficiency, hepatitis B, HIV, and insulin-dependent diabetes mellitus); severe psychiatric disturbances that might interfere with the study protocol; pregnancy; illiteracy; use of antidepressants, anxiolytics, or antipsychotics; and psychological treatment.

### Procedure

**Figure 1:**
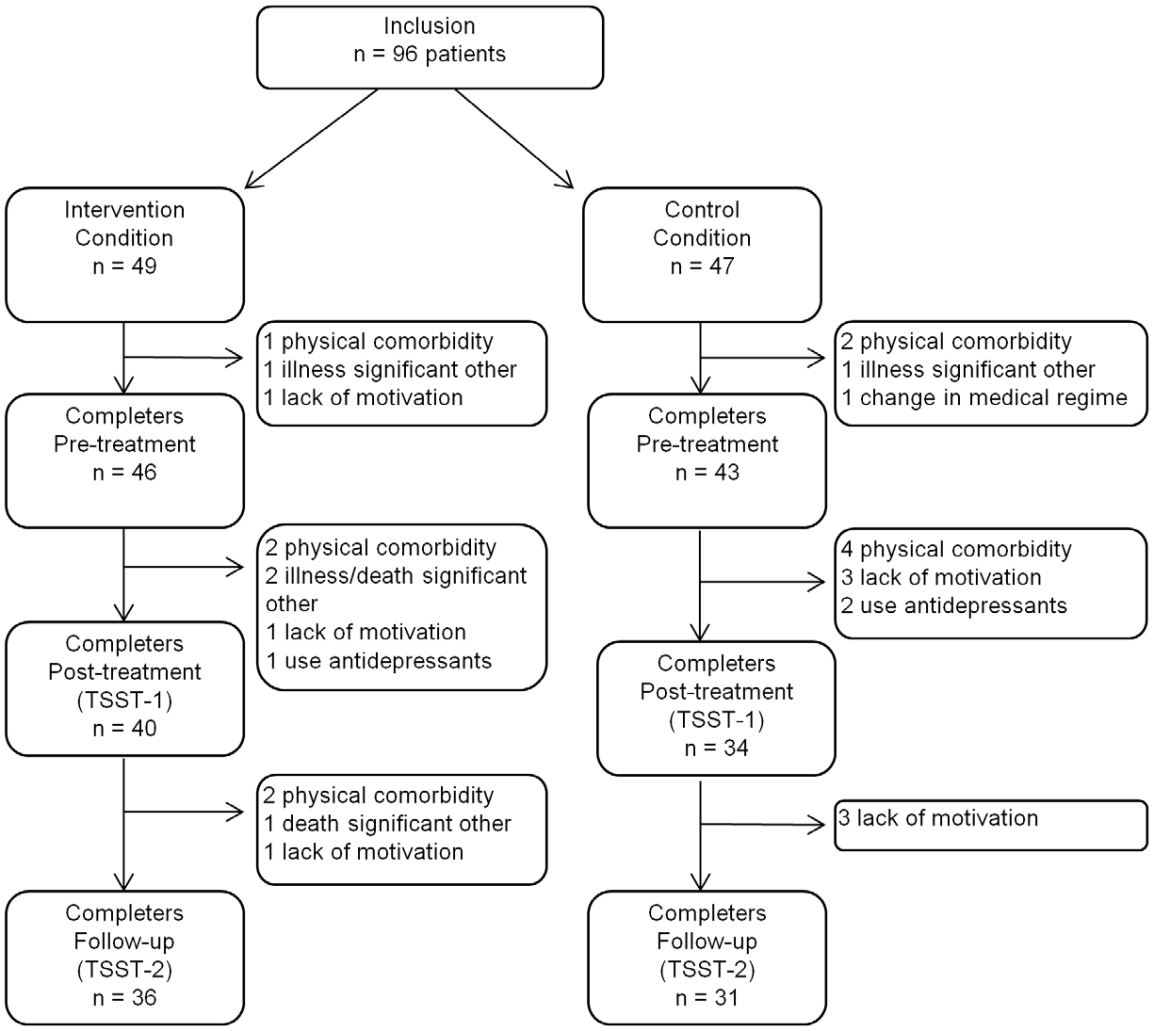
Flow chart showing participant selection and drop-out.

Ninety-six eligible patients were enrolled (see Figure 1) and randomized through simple randomization with an equal allocation ratio to one of two parallel groups, the control or the treatment condition, in accordance with the fixed therapist's time schedule and using a computerized random generator scheme made by an independent researcher. Allocation was concealed for the participant enroller until the moment that participants were scheduled into the treatment program. After randomization, 19 participants (n = 8 intervention, n = 11 control) withdrew from the study prematurely (prior to the first stress test), because of physical comorbidity (n = 3 intervention, n = 6 control), severe illness or death of a significant other (n = 3 intervention, n = 1 control), a change in pharmacotherapy (n = 1 control), or lack of motivation (n = 2 intervention, n = 3 control). In addition, 3 participants (n = 1 intervention, n = 2 control) reported taking antidepressants or anxiolytics after randomization and were excluded based on our predefined exclusion criteria. Seven of 74 participants withdrew from the second stress test (n = 4 intervention, n = 3 control) because of physical comorbidity (n = 2 intervention), death of a significant other (n = 1 intervention), and lack of motivation (n = 1 intervention, n = 3 control). There were no differences in sociodemographic variables (sex, age, education level) and psychological and physical functioning at baseline (anxiety, negative mood, positive mood, Disease Activity Score 28 (DAS28)) between the drop-outs and the completers.

Participants were post hoc divided into 2 subgroups based on the participant's risk status by means of a median split on a composite score of baseline anxiety and negative mood assessed with the IRGL (see Measures) [30], [32].

#### Study design

At the first assessment, the medical history and current disease activity of all participants were evaluated at the University Medical Centre, and in the subsequent two weeks half of the participants started the individual stress management training program. All participants performed a stress test three weeks after the first assessment (i.e., second assessment) and 9 weeks thereafter (i.e., third assessment). Stress test sessions were run between 13.00 and 15.30 hours. Participants were asked to refrain from using caffeine, alcohol, nicotine, or physical exercise on the test day, and from eating 2 hours before the first blood sample was drawn. Forty minutes before the stress test, a venous catheter was inserted into the non-dominant arm (immunological data presented elsewhere) and participants were asked to rest for 20 minutes. They then performed the stress test. During periods of rest, participants looked at a natural history documentary. Psychophysiological parameters (tension, saliva, and blood) were measured at baseline (i.e., after 20 minutes of rest), immediately after the stress test, and 10, 20, 40, and 60 minutes after cessation of the test.

#### Stress task

The Trier Social Stress Test (TSST) is a standardized laboratory stress task that consists of a mock job interview and mental arithmetic in front of an audience. The persons conducting the TSST were unaware of group allocation of the participants. The TSST lasts 15 minutes, including introduction to the job interview and a 5-minute preparation phase, and has repeatedly been found to induce self-reported, neuroendocrine, and autonomic nervous system responses [38].

#### Stress management training

Participants in the intervention group received individual stress management training with a focus on psycho-education and the principles of applied relaxation, including progressive, cue controlled, and differential relaxation [30], [39]–[42]. In addition, patients were taught breathing and visualization exercises. Participants attended 4 individual 1-hour sessions with a trained therapist over 2 consecutive weeks. Patients received an MP3-player with relaxation exercises and, at the end of each session, a training manual containing a summary of the information and stress-reducing techniques introduced in that session. As consolidating homework, participants assessed stress-relevant situations and behaviours in their daily life and used relaxation exercises for 1 hour at least twice a day during the 2 weeks of the stress management intervention. Subsequently, patients were encouraged to continue the homework assignments, to use the relaxation exercises, to focus on long-term goals, and to stick to a relapse-prevention checklist during the 2-month follow-up period.

### Measures

#### Demographic, clinical, and self-report measures at baseline, post-treatment, and follow-up

*Demographic variables* were assessed with a general checklist for age, sex, marital status, education, and medical history. Educational level was measured using seven categories that can be classified as primary, secondary, and tertiary education, representing on average 7, 12, and 17 years of education, respectively.

*Physical functioning* was assessed in terms of disease activity. Disease activity of patients was measured with the DAS28, which is a validated composite score for swelling and tenderness of 28 joints, a Visual Analogue Scale (VAS) of the patients' general health, and the ESR (mm/h) [43].

*Psychological functioning* was measured with the state anxiety and negative and positive mood scales of the IRGL [44], [45]. The IRGL is derived from the Arthritis Impact Measurement Scales (AIMS) [46]. The 10-item anxiety scale is a shortened version of the Dutch State Anxiety Scale [47], [48] and assesses anxiety over the last 2 weeks (sample item: “I worry too much about unimportant matters.”); the 6-item negative mood scale assesses various negative mood states over the previous 2 weeks (sample item: “How depressed were you during the past 2 weeks?”); and the 6-item positive mood scale assesses various positive mood states over the previous 2 weeks (sample item: “How cheerful were you during the past 2 weeks?”).

#### Patients' evaluation of stress management training

After training ended, patients were asked to indicate their satisfaction with the training and its usefulness (score range 0–10, ranging from “not at all” to “very”), and to what extent their distress and tension had improved (score range 1–4, ranging from “not” to “very”).

#### Psychophysiological measures during the stress test at post-treatment and follow-up

*VAS tension.* Participants rated how tense they were on a VAS at baseline (after 20 minutes of rest), during the stress test (retrospectively), and 10, 20, 40, and 60 minutes after cessation of the stress test.

*Alpha-amylase* as a measure of autonomic reactivity. Saliva samples were collected with salivettes (Sarstedt, Rommelsdorf, Germany) and stored at −35°C until further biochemical analyses. After saliva samples were thawed, centrifuged, and diluted, α-amylase (AA) was measured with the Aeroset (Abbott). According to the procedure, α-amylase hydrolyses the reagent CNPG3 (2-chloro-4-nitrophenyl-α-D-maltotrioside) to CPNP (2-chloro-4-nitrophenol), CNPG2 (2-chloro-4-nitrophenyl-α-D-maltoside), maltotriose, and glucose. The rate of CPNP formation was detected spectrophotometrically at 404 nm to give a direct measurement of amylase in saliva.

*Cortisol* as a measure of endocrine reactivity. Salivary cortisol was measured with a commercial Luminescence Enzyme Immunoassay (IBL, Hamburg, Germany). After samples were thawed and centrifuged, 20-µl aliquots of the supernatant were pipetted into anti-cortisol (rabbit-) antibody-coated microtitre plate wells, followed by 100 µl of enzyme conjugate (horseradish peroxydase). After 3-hour incubation at room temperature, the plate was washed and luminescence reagent (luminol/peroxide) was added to each well, with subsequent reading of the signal in a luminometer. At levels of 3.3 and 27.3 nmol/l, within-assay coefficients of variation (CV) were 8.7 and 3.6% respectively, and between-assay CVs were 12.3 and 7.7%. To reduce error variance caused by between-run variation, all samples from one participant were analyzed in the same run.

### Statistics

Analyses were performed on the 74 participants completing the study protocol. Skewed data (i.e., negative mood and all physiological parameters) were logarithmically transformed to render unskewed data distributions before statistical analysis. Between-group differences in age, sex, education, and psychological measures at baseline were tested with independent Student's *t*–tests and Chi-square analyses. For cortisol, the area under the curve (AUCg) was calculated using the trapezoid formula [49]. Baseline differences in psychophysiological outcome parameters (VAS tension, cortisol, and α-amylase) (t = 0 minutes) and AUCg in the intervention and control groups were evaluated with analyses of covariance (ANCOVA). Effects of the stress management training (i.e., psychological/physical functioning and psychophysiological responses to the stress test) were evaluated using a linear mixed model taking into account the specific design features of the study. The primary outcome measure was state anxiety as a measure of psychological distress. The effects on secondary outcomes of psychological and physical functioning (positive and negative mood, and DAS28) and psychophysiological stress parameters (tension, cortisol, and α-amylase measured during the stress test) at the post-treatment and follow-up assessments were also assessed. In analyses of the effects of the stress management training on psychological and physical functioning, measures of psychological and physical functioning were used as dependent variables, and group, baseline measurement of the dependent variable (pretreatment), and time levels (post-treatment and follow-up) were used as independent variables. With regard to the psychophysiological response to the stress test, the three psychophysiological outcome measures (tension, cortisol, and α-amylase) were used as dependent variables, and group, baseline measurement of the dependent variable (t = 0 minutes), and time levels (t = 20; t = 30; t = 40; t = 60; and t = 80 minutes) were used as independent variables. Explorative subgroup analyses were performed to test whether effects were stronger or only held in patients at risk as compared to patients not at risk (also see Procedure) by incorporating risk group and risk group by treatment interactions into the analysis models. A significant interaction was interpreted as an indication of subgroup differences with respect to the effect of the treatment. Stratified analyses were performed to gain a better understanding of the nature of the responses in the subgroups of patients.

For every outcome measure, an unstructured covariance matrix was used to model the dependence between repeated measurements of the dependent variable. Owing to a slightly unequal distribution of sex across the two groups (p = 0.08) and a trend towards higher anxiety scores at baseline in the intervention group (p = 0.09) (see Results section, Patient characteristics), all analyses were performed with the covariates sex and baseline (pretreatment) anxiety. In addition, cortisol analyses were also performed with the additional covariate hormonal contraceptives [36] (see Results section, Patient characteristics).

A priori power calculation resulted in an optimal sample size of N = 64 (expected adjusted effect size of f = 0.45 of the primary outcome measure psychological distress (state anxiety), a power of 0.90, and α = 0.05). However, because there were missing blood samples (a venous catheter could not be inserted in n = 15 patients during one or two stress tests) and the high drop-out rate before the start of the first stress test was high (n = 22; see procedure), we increased the earlier estimate of 64 patients to 96. In total, data of the 74 patients included in the analyses were 95% complete regarding psychological and physical outcomes at baseline, post-treatment, and follow-up, and 97% complete regarding psychophysiological parameters at post-treatment and follow-up. Physiological data for three participants at one of the assessment moments (cortisol levels in two participants and amylase levels in one participant) were excluded from analyses because levels were four standard deviations higher than the mean for at least one of the six time points during the stress test. All analyses were performed using SPSS 16.0 for Windows. For all analyses, the significance level was α = 0.05 (two-sided). Unless indicated, all results are means ± standard deviation (SD).

## Results

### Patient characteristics

Baseline demographic and disease-related characteristics of the 74 participants are presented in Table 1. The two groups did not differ significantly regarding age, education level, mean disease activity, and mean disease duration. However, there tended to be more women in the intervention group (χ^2^ = 3.155, p = 0.08). Thirty-three of 74 patients were taking biologicals (including etanercept, adalimumab, abatacept, and infliximab), 54 patients were taking DMARDS (including methotrexate (MTX), sulfasalazine, hydroxychloroquine, leflunomide, and/or azathioprine), 47 patients were taking NSAIDs, and 14 patients were taking prednisone (<10 mg/day). Twenty-four patients received medication known to affect the ANS (including β-blockers, ACE-inhibitors, Ca^2+^-blockers, α_1_-blockers, thiazides (or –related), ACh-receptor antagonists, β_2_-adrenergics, and anti-histamines), and 7 patients used hormonal contraceptives (6 intervention, 1 control; χ^2^ = 3.120, p = 0.08). There were no significant group differences in the use of biologicals, DMARDs, steroids, and medication known to influence the ANS, except for the use of NSAIDs, which was significantly higher in the intervention group (χ^2^ = 7.349, p = 0.01). There were no significant group differences in pretreatment measures of negative and positive mood, and disease activity, but anxiety scores tended to be higher in the intervention group than in the control group (t(67.835) = −1.715, p = 0.09) (Table 2). Consequently, all further analyses were performed with covariates sex and baseline anxiety, with the additional covariate hormonal contraceptives for endocrine analyses.

**Table 1:** Demographic characteristics, disease severity, and medical regimen of patients with rheumatoid arthritis in the intervention and control groups*.

	Intervention	Control	
	(n = 40)	(n = 34)	p-value
No. females/males	27/13	16/18	.08
Age (years ± SD)	57.2±11.8 (range 24–75)	60.7±9.2 (range 26–80)	.17
Education level (%)			.56
Primary	7.5%	2.9%	
Secondary	60.0%	70.6%	
Tertiary	32.5%	26.5%	
Disease Activity (DAS28)	2.6±1.0 (range 0.8–4.5)	2.6±1.1 (range 0.5–5.1)	.81
Disease duration (years ± SD)	15.7±10.9 (range 5–51)	12.4±7.6 (range 3–37)	.15
No. of patients currently under treatment for RA	38	32	
Biologicals	17	16	.69
DMARDs	31	23	.43
NSAIDs	31	16	.007
Steroids (<10 mg/day)	9	5	.39

*Values are means ± SD. RA = rheumatoid arthritis; DMARDs = disease-modifying anti-rheumatic drugs; NSAIDs = nonsteroidal anti-inflammatory drugs.

**Table 2:** Means (± SEM) and estimated marginal means (± SEM) of psychological and physical outcomes of patients in the intervention condition (IC: n = 40) and the control condition (CC: n = 34) pre- and post-treatment, and at follow-up.

		Means (± SEM)			Estimated marginal means (± SEM)	
		Pre-treatment	Post-treatment	Follow-up	Post-treatment	Follow-up
*Psychological functioning*						
Anxiety	IC	17.69 (0.94)	17.15 (0.76)	16.78 (0.74)	16.28 (0.36)	15.95 (0.39)*
	CC	15.68 (0.70)	16.64 (0.79)	16.06 (0.72)	17.47 (0.39)	17.14 (0.42)
Negative	IC	3.23 (0.66)	2.97 (0.53)	2.17 (0.46)	0.92 (0.06)	0.79 (0.08)
Mood	CC	1.94 (0.40)	2.00 (0.51)	1.77 (0.43)	0.90 (0.07)	0.78 (0.08)
Positive	IC	12.00 (0.68)	12.10 (0.64)	13.00 (0.53)	12.76 (0.48)	13.35 (0.40)*
Mood	CC	12.97 (0.61)	12.18 (0.75)	12.48 (0.64)	11.55 (0.49)	12.14 (0.43)
*Physical functioning*						
DAS28	IC	2.62 (0.16)	2.81 (0.16)	2.51 (0.20)	2.68 (0.09)	2.43 (0.10)
	CC	2.56 (0.19)	2.56 (0.19)	2.48 (0.19)	2.68 (0.09)	2.43 (0.11)

*Significant between-group effect (p≤0.05). Means of outcomes pre- and post-treatment, and at follow-up; and estimated marginal means of post-treatment and follow-up, corrected for pretreatment measures (and other covariates).

### Psychological and physical functioning

#### Satisfaction and usefulness of the training

Patients rated their satisfaction with the intervention with a score of 8.1± SD 1.2 and its usefulness with a score of 7.6± SD 2.0. Approximately 87% of patients in the intervention group reported an improvement in stress and tension after the training (little improvement by 42%, moderate improvement by 32%, and strong improvement by 13%).

#### Psychological functioning in intervention and control condition

Means and estimated marginal means (EMM; i.e., means corrected for the covariates) (± SEM) of the psychological and physical outcomes are presented in Table 2. A significant group effect was found for anxiety (F(1,69.887) = 5.579, p = 0.02); the intervention group had a significantly lower anxiety score than the control group after the intervention. Furthermore, patients in the intervention group had significantly higher levels of positive mood after the intervention than did patients in the control group (group effect, F(1,67.436) = 4.851, p = 0.03). No overall group effect was observed for negative mood (F(1,68.389) = 0.028 p = 0.87). Subgroup analyses showed a significant interaction effect between condition (intervention/control) and risk group (high/low) for anxiety (F(1,68.002) = 7.820, p<0.01) and negative mood (F(1,66.893) = 11.509, p<0.01), but not for positive mood (F(1,65.985) = 0.205, p = 0.65), indicating that high-risk patients responded differently to the stress management training with regard to anxiety and negative mood than did low-risk patients. Inspection of the data by post-hoc tests revealed that lower anxiety scores (group effect, F(1,32.725) = 8.128, p<0.01) and lower negative mood scores (F(1,31.473) = 4.021, p = 0.05) were present in the subgroup of high-risk patients in the intervention group compared to high-risk controls, but not in low-risk patients (group effect anxiety, F(1,33.898) = 0.019, p = 0.89; reverse group effect negative mood, F(1,31.677) = 8.644, p<0.01). In addition, a trend towards higher positive mood scores was observed in high-risk patients in the intervention group compared to controls (F(1,31.578) = 3.548, p = 0.07), but not in low-risk patients (F(1,31.256) = 0.691, p = 0.41).

#### Physical functioning in intervention and control condition

There were no differences in disease activity (DAS28) between control and intervention groups after the stress management intervention (F(1,61.610) = 0.004, p = 0.95). Subgroup analyses showed no interaction effect between condition (intervention/control) and risk group (high/low) (F(1,59.864) = 0.051, p = 0.82).

### Psychophysiological stress reactivity

#### Stress manipulation check

**Figure 2:**
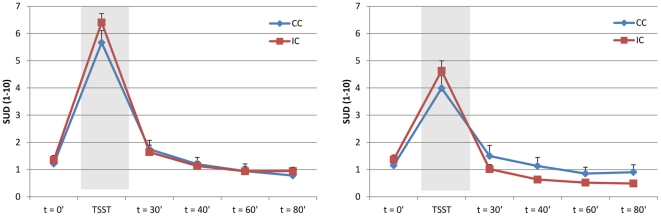
Self-reported response to stress. Mean stress-induced VAS tension levels (± SEM) in the intervention (IC) and control (CC) conditions post-treatment (left; IC, n = 40; CC, n = 34) and at follow-up (right; IC, n = 36; CC, n = 31).

**Figure 3:**
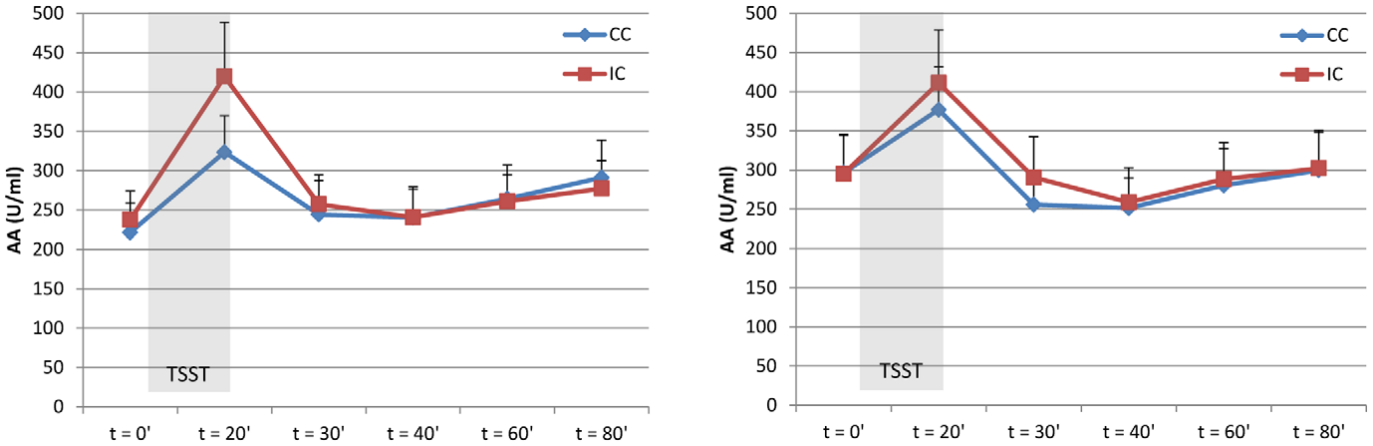
Autonomic response to stress. Mean stress-induced α-amylase levels (± SEM) of patients in the intervention (IC) and control (CC) conditions at post-treatment (left; IC_AA_ = 9; CC_AA_ = 3) and at follow-up (right; IC_AA_ = 35; CC_AA_ = 31).

**Figure 4:**
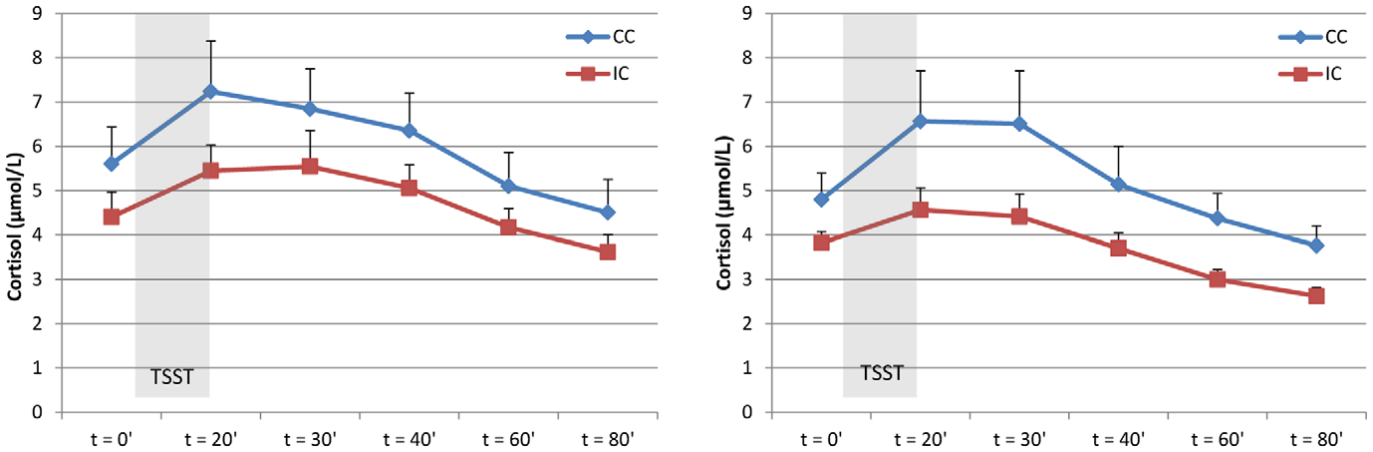
Endocrine response to stress. Mean stress-induced cortisol levels (± SEM) in the intervention (IC) and control (CC) conditions post-treatment (left; IC, n = 39; CC, n = 32) and at follow-up (right; IC, n = 34; CC, n = 31).

Both after treatment and at follow-up, the stress test induced a significant increase in tension (time effect, F(1,73) = 304,899; p<0.001, and F(1,66) = 182.031, p<0.001, respectively; Figure 2), α-amylase (time effect, F(1,69.211) = 46.003; p<0.001, and F(1,65) = 21.404, p<0.001, respectively; Figure 3), and cortisol levels (time effect, F(1,69.041) = 29.566; p<0.001, and F(1,63.003) = 9.688, p<0.01, respectively; Figure 4) in all patients.

#### Baseline differences between intervention and control condition

Both after treatment and at follow-up, there were no significant differences between the intervention and control groups in baseline levels (t = 0 minutes) of tension (F = 0.230, p = 0.63 and F = 0.444, p = 0.51, respectively), α-amylase (F = 0.007, p = 0.93 and F = 0.326, p = 0.57, respectively) and cortisol (F = 1.530, p = 0.22 and F = 1.729, p = 0.19, respectively).

#### Post-treatment psychophysiological stress reactivity

After treatment, levels of self-reported tension in response to the stress task were similar in the intervention and control groups (group effect, F(1,69.000) = 0.340, p = 0.56, Figure 2), as was autonomic reactivity (group effect α-amylase, F(1,66.359) = 0.068, p = 0.80, Figure 3), and endocrine reactivity (group effect cortisol, F(1,64.287) = 0.315, p = 0.58, Figure 4; and AUCg: F(1,66) = 0.734, p = 0.40, Table 3), indicating that patients in the intervention group did not have an altered psychophysiological response to stress compared to patients in the control group after the intervention. Subgroup analyses also showed no interaction effect between condition (intervention/control) and risk group (high/low) for psychophysiological measures of stress, indicating that high-risk and low-risk patients did not respond differently to the stress management training with regard to stress-induced levels of tension, α-amylase, and cortisol.

**Table 3:** Area under the curve (AUCg) for cortisol (means ± SEM) in the intervention (IC) and control (CC) conditions post-treatment and at follow-up.

	Post-treatment	Follow-up
Intervention condition	42.59 (4.50)	33.46 (2.73)
Control condition	54.04 (7.30)	47.05 (6.96)

#### Follow-up psychophysiological stress reactivity

At the follow-up assessment, self-reported tension elicited by the stress test was significantly lower in patients in the intervention group than in patients in the control group (group effect, F(1,62.000) = 6.092, p = 0.02, Figure 2). In addition, there was a significantly diminished cortisol response (group effect, F(1,59.010) = 4.877, p = 0.03, Figure 4) and a trend towards a lower total cortisol output (AUCg) in the intervention group compared with the control group (AUCg, F(1,60) = 3.689, p = 0.06, Table 3). The autonomic response was similar in the two groups (group effect α-amylase, F(1,61.085) = 0.301, p = 0.59, Figure 3). Subgroup analyses showed no interaction effect between condition (intervention/control) and risk group (high/low) for tension (F(1,60.000) = 1.919, p = 0.17), but a trend towards an interaction effect for α-amylase (F(1,58.996) = 2.752, p = 0.10) and cortisol (F1,57.100) = 3.682, p = 0.06), indicating that high-risk patients tended to respond differently to the stress management training with regard to physiological measures of stress than did low-risk patients. Inspection of the data by post-hoc tests revealed that high-risk patients in the intervention group had or tended to have lower overall levels of tension, α-amylase, and cortisol than did high-risk patients in the control group (group effect tension, F(1,28.000) = 6.768, p = 0.02; group effect α-amylase, F(1,28.052) = 3.495, p = 0.07; group effect cortisol, F(1,25.384) = 7.450, p = 0.01; and AUCg F(1,27) = 5.264, p = 0.03); this was not the case for the low-risk patients (group effect tension, F(1,29.000) = 1.965, p = 0.17; group effect α-amylase, F(1,28.000) = 1.277, p = 0.27; group effect cortisol, F(1,27.000) = 0.818, p = 0.37; and AUCg (F(1,28) = 0.548, p = 0.47).

## Discussion

This is the first study to assess psychological functioning and psychophysiological responsiveness (subjective, autonomic, and neuroendocrine) to a psychosocial stress task in patients with RA who had received training in stress management. Results indicated high satisfaction and perceived usefulness of the training, and a lower anxiety and higher positive mood after the training in the stress management than in the control group. No effect on disease activity or post-treatment psychophysiological stress responsiveness was found, but at follow-up (9 weeks after the training) the stress management group showed a lower tension and cortisol response to stress than the control group. These results were particularly evident in a subgroup of patients psychologically at risk, supporting previous findings of increased treatment effects in at-risk patients [32], [50]. Results of this study suggest that short-term individual stress management training is not only able to improve psychological functioning by the level of tension, but may also alter psychophysiological responses to stress by reducing levels of cortisol.

Stress might have detrimental effects on health, particularly in clinical populations. Over the last decade, there has been an increasing interest in the physiological effects of stress management interventions for patient groups [15]–[21]. Studies of various forms of stress management or cognitive-behavioral therapy in patients with RA have only incidentally reported changes in overall disease activity or biological indicators of disease after the intervention, such as a decrease in overall disease activity [51], [52], self-reported disease flare-ups [24], and joint tenderness [53] in the intervention group compared with the control group. Changes in cortisol values [54], cytokine INF-γ [54], C-reactive protein [28], and ESR [51] have also been reported. In a response to the aforementioned studies, the current study uniquely investigated the effects of a stress management intervention on the acute-phase physiological response to stress. It seems apparent that alterations on the physiological level might particularly occur when interventions are successful in changing the appraisal or perception of stressors [55]. We found that anxiety was significantly, but modestly, reduced after 2 weeks of individual stress management training. After an interval of 9 weeks, during which participants practiced the stress management exercises at home, focusing on long-term stress management and relapse prevention, stress-induced tension was slightly lower and there was a lower stress-evoked cortisol response in the intervention group compared with patients in the control group. The effect of stress management training on psychophysiological stress responsiveness appears to be delayed, possibly because repeated exercise during two months might have stronger effects than exercise of two weeks; it takes time to integrate the learned exercises into the daily lives of participants and to help them cope with stress-provoking situations. Results are in line with preliminary evidence suggesting that intervention-related physiological changes, particularly those related to the immune system, might become more pronounced with time [52], [56].

To our knowledge, only two other studies assessed the acute-phase physiological response to a laboratory stressor after stress management [35], [36]. Healthy males participating in a group-based cognitive-behavioral stress management training showed a significantly diminished cortisol response to the TSST 2 weeks after the intervention [35], and this pattern, although less pronounced, was also observed 4 months after a similar training in male and female subjects [36]. Our results provide preliminary evidence that, in line with recent findings in healthy populations, stress management might also alter endocrine responsiveness to a stress task in a clinically comprised population of patients with RA. Our findings on endocrine responsiveness extend recent results suggesting that basal cortisol levels and stress-induced cortisol reactivity in patients with RA might not be significantly different from those of healthy participants [8], [57]. This implies that the endocrine stress response system could be a target for stress management interventions not only in healthy subjects, but also in patients with immune-mediated diseases such as RA. These interventions might prevent the possible negative physiological consequences of stress on health. Although a reduced psychophysiological stress reaction was found at the follow-up in the stress management group as compared to the control group, this was not accompanied by a simultaneous decrease in disease activity. Because the psychophysiological results were only found at the longer term, this could imply that the effects on disease activity may have occurred even later. Theoretically, a lowered cortisol response might reflect a decreased psychological stress level and/or an improvement in the functioning of all physiological regulatory systems [e.g., 54]. However, no studies have yet reliably shown the consequences of non-pharmacological cortisol changes in rheumatoid arthritis and future studies with a longer-term follow-up are needed to provide insight into this question.

In contrast to altered responses on self-reported tension and cortisol, autonomic reactivity to stress was similar in the two patient groups, as evidenced by the similar levels of α-amylase levels in saliva, an indicator of sympathetic activity [58], [59]. The stress management intervention included principles and techniques that are mainly aimed at reducing tension and negative emotion by inducing a generalized relaxation response [60], which is hypothesized to dampen sympathetic activity [61]. Several studies investigating the effects of relaxation on autonomic changes at baseline or in stress-provoking situations have reported reduced galvanic and cardiovascular reactivity, but evidence of altered autonomic responsiveness is not unequivocal [11]–[13], [62]–[64]. Our results suggest that the responses of the ANS and HPA axis to (repeated) stress are not necessarily synchronous; a phenomenon that has also been documented after recurrent exposure to the same stressful stimulus, both in animal and human research [65]. Whereas (social-evaluative) threat and uncontrollability might be the most important components contributing to an endocrine response to a laboratory stressor [66], autonomic reactivity could be an a-specific response to more generalized arousal, such as the effort to do well [67], [68]. As the cortisol response to a stressor is sensitive to emotions and appraisals that are associated with threats of the social self, such as rumination and submissiveness [69], we hypothesize that the training specifically influenced the endocrine response to stress due to changes in specific emotions.

Overall, subgroup analyses showed that the effects of the stress management training on specific psychological outcomes and physiological stress responses (anxiety and cortisol levels) were particularly evident in a subgroup of patients at risk. Previous studies have shown that particularly patients with RA with heightened levels of anxiety and depression benefit from cognitive-behavioral therapy, not only after treatment but also at follow-up assessments [32]. The importance of subgroup analyses has also been acknowledged in other patient populations [70]–[72]. The lower anxiety and cortisol levels that were observed in the intervention group at follow-up might be attributed to the subgroup of high-risk patients. Additional subgroup effects were found for negative mood and α-amylase levels at follow-up in the subgroup of high-risk patients only. The latter findings support the idea that beneficial effects of treatment might be particularly observed in dysfunctional groups of patients and highlights the importance of identifying subgroups of patients most likely to benefit from a specific intervention in future studies of stress.

This study has several limitations. First, exclusion criteria with regard to physical and psychological comorbidity may have resulted in a homogenous sample of patients showing relatively mild disease activity at baseline. In addition, the sample size was relatively small for the subgroup analyses, particularly when considering multiple testing. Therefore, the results of this study, particularly those regarding subgroups of patients, should be interpreted with caution and should be replicated in larger groups of patients. Secondly, there were marginal baseline differences between the intervention and control groups, with a trend towards a higher female-to-male ratio in the intervention group and higher anxiety scores. We statistically controlled for differences by adding these confounders as a covariate in all analyses, in addition to the use of oral contraceptives for endocrine analyses. It is well-documented that not only has a person's sex differential effects on physiological stress response patterns [73], [74], but also the menstrual cycle, menopause, and the use of oral contraceptives of females influence the cortisol response to laboratory stress paradigms [75], which makes it difficult to control for these effects in a heterogeneous group of patients with arthritis. Thirdly, due to the character of the study, which included a no-treatment control condition, it was impossible to blind patients and researchers for the treatment status of the participants. However, by blinding the persons conducting the Trier Social Stress Test for the treatment status of participants, we tried to limit possible bias on the psychophysiological stress response as much as possible. Lastly, we decided against pre- and post-treatment assessment of psychophysiological stress reactivity, because repeated exposure to the stress test has been found to elicit small habituation effects [76], [77]. In addition, the small effects found on psychophysiological measures at the follow-up assessment might have been larger if the stress test would have been performed only once, at the follow-up assessment.

This is the first study to provide preliminary evidence that a relatively short stress management intervention not only improves psychological functioning, but may also influence the psychophysiological response to stress (self-reported tension and cortisol reactivity) in patients with RA, particularly those psychologically at risk. Our study highlights the need to look at individual differences in stress responsiveness and psychological factors that are able to influence stress response patterns. Interventions such as the current stress management training, alone or as a part of a more comprehensive treatment programme, may prove useful in preventing the detrimental effects of stress on patients with systemic inflammatory diseases, such as RA.

## Supporting Information

Checklist S1 CONSORT Checklist.
(DOC)

Protocol S1 Trial Protocol.
(DOC)
