# Supplementary material for: Psychophysiological Responses to Stress after Stress Management Training in Patients with Rheumatoid Arthritis
Source: PLoS One. 2011 Dec 6;6(12):e27432. doi: 10.1371/journal.pone.0027432 (PMC3232221; doi:10.1371/journal.pone.0027432)
Supplement: Protocol S1 — Trial Protocol. (DOC) [file pone.0027432.s002.doc]

|  | | | | NR | | | **06** | | | **-** | **I** | | | **-** |  | |  | |
| --- | --- | --- | --- | --- | --- | --- | --- | --- | --- | --- | --- | --- | --- | --- | --- | --- | --- | --- |
| GRANT REQUEST FOR SCIENTIFIC RESEARCH | | | | | | | | | | | | | | | | | | |
| **1** | | **General information** | | | | | | | | | | | | | | | | |
|  | | Project leader | | Ms. Dr. A.W.M. Evers | | | | | | | | | CV attached: | | | | | yes |
|  |  | Department | | Medical Psychology 840 | | | | | | | | | | | | | | |
|  |  | Institute | | Radboud University Medical Centre Nijmegen | | | | | | | | | | | | | | |
|  |  | Address | | P.O. Box 9101 | | | | | | | | | | | | | | |
|  |  | Postcode/city | | 6500 HB Nijmegen | | | | | | | | | | | | | | |
|  |  | Phone | | 024-3613608 | | | | | | | | | | | | | | |
|  |  | Fax | | 024-3613425 | | | | | | | | | | | | | | |
|  |  | E-mail | | [a.evers@mps.umcn.nl](mailto:a.evers@mps.umcn.nl) | | | | | | | | | | | | | | |
|  | | | | | | | | | | | | | | | | | | |
| **2** | | **Study title** | | | | | | | | | | | | | | | | |
|  | | **Psychophysiological stress mechanisms in rheumatoid arthritis** | | | | | | | | | | | | | | | | |
|  | | | | | | | | | | | | | | | | | | |
| **3** | | Project characteristics | | | | | | | | | | | | | | | | |
| Bij pilot project dit gaarne vermelden.  Please note if it’s a pilot project | | 1. Classification | | | | | | Duration in years | | | | | | | | | | |
|  |  | Experimental and prospective | | | | | | 3 | | | | | | | | | | |
|  |  | 1. Start and end date | | From: | 1-09-2006 | | | To: | | | | 1-09-2009 | | | | | | |
|  | | | | | | | | | | | | | | | | | | |
| **4a** | **Participants** | | Will research be done on humans? | | | | | | | | | | | | | yes | | |
| Indien METc goedkeuring reeds is verkregen toestemmingsbrief aub meesturenAttach letter of approval if IRB approval has been obtained | | | Healthy participants | | | No | | | Number | | | | | | | 0 | | |
|  |  |  | Patients | | | Yes | | | Number | | | | | | | 64 | | |
|  |  |  | Ethical review (IRB/METc) | | | | | | Positive / partly not yet reviewed | | | | | | | | | |
| **4b** | **Laboratory animals** | | Are experiments planned with animals? | | | | | | | | | | | | | no | | |
| Indien DEC goedkeuring reeds is verkregen toestemmingsbrief aub meesturenAttach letter of approval if approval for animal experiments has been obtained | | | species | | | | | |  | | | | | | | | | |
|  |  |  | number per year | | | | | |  | | | | | | | | | |
|  |  |  | Ethical review (DEC) | | | | | |  | | | | | | | | | |

| **5** | 1. **Summary (laymen’s language) DUTCH version** |
| --- | --- |
| Omvat de algemene probleemstelling, waarom het onderzoek wordt uitgevoerd, wat en/of wie wordt onderzocht, op welke manier, en wat de te verwachten resultaten zijn. In voor *leken* begrijpelijke taal, zowel een Nederlandse als een Engelse versie. De samenvattingen moeten binnen de aangegeven kaders passen! | De veelbelovende medicamenteuze behandelingen die de laatste decennia werden ontwikkeld voor reumatoïde artritis (RA) blijken bij een deel van de patiënten onvoldoende werkzaam te zijn. Reumatologen en patiënten schrijven dit mede toe aan psychologische stress. Ook in onderzoek werd ondersteuning gevonden voor de veronderstelling dat stressfactoren een inflammatoire aandoening als RA kunnen beïnvloeden. Tevens zijn er aanwijzingen dat immunologische en neuroendocriene veranderingen voor dit verband verantwoordelijk zijn. De tot op heden verkregen bevindingen uit onderzoek - dat veelal methodologische tekort-komingen kent - zijn echter inconsistent, vooral met betrekking tot immunologische en neuro-endocriene veranderingen. Wel werden bij RA duidelijke aanwijzingen gevonden dat stress mechanismen vooral een rol spelen bij patiënten met een psychologisch risicoprofiel voor stress (d.w.z een verhoogde stressgevoeligheid). In het huidige project zal deze hypothese experimenteel verder worden onderzocht. Het project sluit aan bij een lopend prospectief onderzoek waarin 100 RA patiënten 6 maanden met wekelijkse en maandelijkse psycho-fysiologische metingen worden gevolgd (cofinanciering NWO-Veni, UMCN). In het huidige project zal bij een subgroep van 64 RA patiënten (32 high risk en 32 low risk) het effect worden onderzocht van een experimentele stresstaak en een kortdurende stress-management interventie. Het valt te verwachten dat - in aansluiting pilot-resultaten van de prospectieve studie - enkel bij risicopatiënten de stresstaak en de stressmanagement interventie effect hebben op specifieke zelfgerapporteerde, immunologische en neuroendocriene stressindicatoren, die op hun beurt van invloed zijn op de ontstekingsactiviteit en lichamelijke klachten, zoals pijn. Dit project maakt het mogelijk bij eenzelfde cohort patiënten een prospectieve en experimentele benadering te combineren waardoor inzicht verkregen wordt in de invloed van korte- en lange-termijn stressoren en stress-management op het psycho-fysiologische stressrespons-systeem in RA. Verwacht wordt dat hiermee een robuust model voor psychofysiologische stress mechanismen ontwikkeld kan worden dat bijdraagt aan de diagnostiek en behandeling bij RA en andere chronisch inflammatoire aandoeningen. |
|  | 1. **Summary (laymen’s language) ENGLISH version** |
| Concerns the general research focus, reason for research, what or who will be researched, in what way, and the expected results. In laymen’s language. The summaries may not exceed the given space. | In spite of the promising developments for pharmacological treatments in rheumatoid arthritis (RA) during the last decennia, there are still a substantial number of patients for whom pharmacological treatments have an insufficient effect. Both rheumatologists and patients attribute this treatment failure partly to the effects of psychological stress factors. Also in empirical research, there is increasing evidence for the idea that psychological stress factors can influence chronic inflammatory diseases, such as RA. These relationships are assumed to be (at least partly) mediated by immune and neuroendocrine function. Despite preliminary evidence, the limited research - which is frequently characterized by methodological short-comings - revealed inconsistent findings, particularly regarding immune and neuroendocrine mediation. In addition, our and other previous work suggests that stress mechanisms affecting disease activity and immunological parameters are primarily evident in patients with a psychological risk profile for stress. In line with these findings, the present experimental project is an extension of a prospective study with comprehensive psychophysiological assessments (weekly and monthly) during 6 months in 100 RA patients (co-financing NWO-Veni, UMCN). As a follow-up to the prospective study, the effects of an experimental stress-task and a short-term stress-management intervention are investigated in a subgroup of 64 RA patients (32 with high risk and 32 with low risk). In line with pilot results from the prospective study, it is expected that, only in patients at risk, the experimental stress task and the stress-management intervention affect self-reported, immune and neuroendocrine indicators of stress-reactivity, which in turn affect disease activity and physical symptoms of pain. The combination of the experimental and prospective designs in the same patient cohort strengthens the study of various shorter-and longer-term stressors and of a stress-management intervention for the psychophysiological stress-response system in RA patients. It can be expected that this approach will result in a robust model of psychophysiological stress mechanisms that contributes to improved diagnosis and treatment of RA and other chronic inflammatory diseases. |

| **6** | 1. **Theoretical background** |
| --- | --- |
| Geef hier duidelijk aan op basis waarvan het projectvoorstel is opgesteld. Zonder duidelijke theoretische achtergrond maakt een project in de regel weinig kans (behalve pilot projecten). De relevante literatuur wordt onder 6d vermeld.  Please expand on the theoretical background for the project. Without a clear theoretical background a project usually will not be granted (pilot-projects excluded). The relevant publications should be listed under 6d. | Various research lines suggest a relationship between stress factors and the course of chronic inflammatory diseases, such as rheumatoid arthritis (RA). For example, immune processes together with autonomic nervous and endocrine (HPA axis) systems play a role in the course of RA. There is also increasing evidence that psychological stressors and risk factors can affect the course of RA, and these relationships are supposed to be at least partly mediated by immune and neuroendocrine function (Ader et al, 2001; Straub et al., 2005). More specifically, prospective studies on RA indicate that psychological stressors (specifically interpersonal stressors) and risk factors (specifically passive-avoidant coping, perceived helplessness and lack of social support) affect future diseases activity (ESR and joint scores) and pain (self-report and joint scores) (e.g, Evers et al., 1998, 2001, 2003a/b, Zautra et al., 1997, 1998, 2004). Preliminary relationships between these psychological factors and specific immune and neuroendocrine parameters (e.g,. IL-1, IL-6, TNF alpha, cortisol) in experimental and cross-sectional studies or stress-management studies further suggest a mediating role for specific immune and neuroendocrine parameters (e.g., Hirano et al., 2001; Matzuzaki et al., 2006; Richards, 2005; Zautra et al., 2004). Notwithstanding this preliminary evidence, research has been inconsistent, particularly for immune and neuroendocrine mediation (Ader et al., 2001; Straub et al, 2005; Zautra, 2003). Different reasons have been attributed to this inconsistency:   1. *Patients psychologically at risk:* In addition to methodological and measurement issues (see point 2), our work and the work of others clearly indicate that the lack of consistent evidence can be attributed to patients’ variability with regard to psychological risk factors (e.g, Evers, 2001a/b, 2002, 2003a/b; Zautra, 1998, 2004). Research on psychophysiological stress mechanisms has generally neglected patients’ interindividual variability regarding the psychological risk factors (see Segerstrom, 2001; Huyser & Parker, 1999; Ligier & Sternberg, 2001). More specifically, there is increasing evidence that psychophysiological stress mechanisms of altered stress reactivity responses are primarily evident in patients psychologically at risk. 2. to have enduring effects on disease activity and accompanying physical symptoms (e.g, Evers et al,2001a/b,2002,2003a/b; Zautra et al., 1998,2004; Richards, 2004). More recently, there is also support that specific immune and neuroendocrine parameters mediate this relationship for patients psychologically at risk. Specifically, support has been found that altered IL-6 and cortisol-levels play a central role in this relationship for patients psychologically at risk (Maas; 2001; Richards et al, 2005; Zautra et al., 2004).   *2) Psychophysiological assessments:* In addition to lack of attention for psychological risk factors, most studies in this upcoming field are characterized by methodological problems, e.g. small N, cross-sectional designs and limited psychophysiological stress parameters. Until now, a comprehensive set of psychological, immune and neuroendocrine parameters in a methodo-logically sounded design has rarely been studied. In addition, mediation of neuroendocrine and immune function have hardly been tested over time (and only with small N), and not in combination with experimental approaches, which is a prerequisite to study effects of acute and longer-term stressors on various clinically relevant outcomes of inflammation and immune and neuroendocrine function (Straub et al., 2005; Ligier & Sternberg, 2001). Specific immune and neuroendocrine parameters that are known to be crucial to both chronic inflammation and psychophsyiological stress mechanisms, such as cytokines IL-1,IL-6, TNF-alpha, in addition to indicators of HPA axis function, particularly cortisol, have only incidentally been studied in RA (e.g, Zautra, 2004; Dekkers, 2001,2003). Of special interest are also recent key parameters in the RA immuno-neuroendocrine interface, such as MIF and IL-18, which act closely with glucocorticoids to regulate the immune and inflammatory response (e.g. activating expression of IL-1, IL-6, TNF-alpha) (LeBlanc, 2006; Liew, 2003; Radstake et al., 2005).  In conclusion, a comprehensive research approach, combining prospective and experimental approaches on psychophysiological stress mechanisms in RA that focuses on key immuno-neuroendocrine parameters and patients psychologically at risk, has not yet been conducted. Research that integrates a combined prospective and experimental approach - to study the effects of short- and longer term stressors as well as the therapeutic possibilities to alter psychophysiological stress reactivity responses in patients psychologically at risk - is a prerequisite for the development of a comprehensive psychophysiological stress model in RA. |

| **6** | 1. **Preparatory work** |
| --- | --- |
| Geef hier duidelijk aan welk eigen voorwerk er is verricht. Zonder voldoende voorwerk is een meerjarig project niet mogelijk en kan alleen een 1-jarig pilot project worden ingediend.  De relevante literatuur wordt onder 6d vermeld.  Please explain the amount of own preparatory work that has been done. Without adequate preparatory work an extended project will not be granted, only 1-year pilot projects.  The relevant publications can be listed under 6d. | There is a longstanding resarch tradition at the Department of Medical Psychology of the UMC St Radboud that focuses on psychophsyiological stress mechanims in RA.  In collaboration with department of Rheumatology of the UMC St Radboud (Prof. dr. P.L.C.M. van Riel), the St Maartenskliniek (Dr. W. van Lankveld) and the Rijnstate Hospital (dr. A.J.L. de Jong) as well as the department of Rheumatology and Clinical Immunology of the UMC Utrecht (Prof. dr. J.W.J. Bijlsma), several studies were performed to clarify the role of psychological risk factors in the course of inflammation and physical functioning of patients with RA. In this line of research, instruments for health status assessments and psycholgocial risk factors were developed (Evers et al., 1998, 2001a/b; Kraaimaat et al, 1997; Kraaimaat & Evers, 2003). In addition, cross-sectional and prospective studies were undertaken to investigate the role of psychological risk factors, such as passive-avoidance factors and helplessness, for the short-term and long-term course of RA (Evers et al., 1997, 1998a, 2001a/b, 2002a/b; Kraaimaat et al., 1995a/b; 2003). Specifically, psychological risk factors were shown to be predictive for the short and long-term course of inflammatory activity and physical functioning in RA and other chronic inflammatory diseases (Evers et al., 1997, 1998, 2001a/b, 2002, 2003a/b; Kraaimaat & Evers, 2003). Recently, this research line has been extended to experimental laboratory stress inductions to study the psycho-physiological stress mechanisms in RA and other chronic pain conditions more closely for patients at risk (van Laarhoven et al, 2005). As a result of this research line, a diagnostic instrument to screen for RA patients psychologically at risk was developed and validated (Evers et al, 2002), an instrument that is currently regularly used in rheumatological practice.  Besides the experimental and prospective research on risk factors, psychological interventions, including generalized treatment for unselected RA patients (Kraaimaat et al., 1995) as well as specific tailor-made intervention for RA risk patients (Evers et al., 2002) were developed and evaluated. While the generic intervention failed to demonstrate effects on physical functioning and immune parameters (Kraaimaat et al., 1995), the effectiveness was demonstrated for a tailored intervention aimed at the modification of psycholgoical risk factors in patients with RA (Evers et al., 2002). At this moment, a similar appraoch is under study and has already proved to be promising in other chronic pain conditions (Evers et al., 2005).  This research line on experimental and prospective research in RA and other chronic inflammatory conditions is currently continued by a comprehensive prospective study examining psychophysiological stress mechanisms in patients psychologically at risk (NWO-Veni; see working plan for a more detailed description). A broad network of excellent national and international experts in the field of RA immunology (prof. P. van Riel, dr T. Radstake), endocrinology (prof. F. Sweep) and psychoneuroimmungology (prof. A. Zautra) are collaborating in this project. Recently, pilot data of 25 RA patients have been analysed, showing that stress-disease relationships are particularly found in patients psychologically at risk. Parallel to the research on psychophysiological stress mechanisms, the department of Rheumatology of the UMC St Radboud has an excellent research history regarding specific innovative immunological findings in rheumatoid arthritis (e.g., MIF, IL-18), recently resulting in another NWO-Veni grant for one of the project members (dr. T. Radstake).  Based on the preparatory work together with the national and international collaborations, the present project is a further logical step in this research line, by making use of a combined prospective and experimental stress-study and stress-management approaches in the same patient cohort - a unique possibility to clarify basic psychophysiological stress mechanisms for RA. |

| **6** | 1. **Goal or hypothesis** |
| --- | --- |
| Geef hier tenslotte aan hoe op grond van de in 6a en 6b gegeven informatie de hypothese tot stand gekomen is.  Please explain how, based on the rationale given in 6a and 6b, the hypothesis for this project has been developed. | The goal of the present study is to get a greater insight into psychophysiological stress mechanisms in RA, by using an unique approach of combining prospective and experimental studies, with a comprehensive set of clinical and self-report measures of psychological, immune and neuroendocrine assessments. Specifically, the prospective field study, for which co-financing has already been received (NWO-Veni, UMCN), makes it possible to study the effects of longer-term, real life stressors on inflammation processes and immune and neuroendocrine function. For this purpose, a group of 100 RA patients are followed during 6 months with weekly (self-report) and monthly (clinical and self-report) assessments. As an extension of this prospective part, the present project focuses on two experimental approaches in randomly selected subgroups of 64 RA patients (32 high vs. 32 low risk patients), to experimentally study the effects of acute stressors and stress-reducing interventions on the stress-reactivity response system of patients with RA. Specifically, patients will be asked to participate in a randomized trial of a short-stress-management intervention (8 sessions during 4 weeks with intensive homework assignments) as well as stress-experiments (1hr) at 3 assessments points (at 0, 4 and 16 weeks; corresponding to the assessments points at pre- and post-treatment and the 3-months follow-up of the stress-management intervention). Clinical, laboratory and self-report assessments of the stress reactivity responses are measured at these assessment points (also see figure p.7).  In line with the preliminary results of the prospective part of the study and recent empirical findings on psychophysiological stress mechanisms (Mason, 2001; Richards et al., 2005; Zautra, et al. 2004), three main hypotheses are investigated:   1. Stress-experiment: During the stress-experiment at first assessment, it is expected that the experimental stressor will have a stronger effect on the *stress reactivity responses* (i.e. self-reported distress, autonomous responses and cortisol levels) for patients at risk than for patients not at risk. 2. Stress-management-intervention:   a. For the stress-management intervention, it is expected that the intervention will have a stronger effect on the *stress-reactivity responses* (i.e. self-reported distress, autonomous responses and cortisol levels) *during the stress-experiment* at post-treatment and follow-up assessment for patients at risk than for patients not at risk.  b. For the stress-management intervention, it is further expected that the intervention will have a stronger effect on *basal responses* of self-reported distress, immune and neuroendocrine responses at post-treatment and disease activity and physical symptoms at follow-up assessment for patients at risk than for patients not at risk.    The comprehensive research design, combining prospective and experimental approaches on psychophysiological stress mechanisms that focus on key immuno-neuroendocrine parameters and patients psychologically at risk, offers a unique opportunity to validate a psychophysiological stress model and the mediation of neuroendocrine and immune function for these relationships in RA. The main innovative aspects are the focus on patients psychologically at risk to clarify relationships of psychological stress factors in RA and to test mediating effects of immune and neuroendocrine parameters in these relationships.  This innovative approach – combining a prospective study with repeated measurements with an experimental stress-condition and stress-management approach in the same patient cohort - enables a generic model of psychophysiological stress mechanisms in RA to be studied. A better understanding of these psychophysiological stress mechanisms will ultimately contribute to diagnosis and treatment of RA and other chronic inflammatory diseases. |

| **6** | 1. **Relevant publications** |
| --- | --- |
| Relevante literatuur van eigen én andere onderzoeksgroepen.  Alleen reviews opgeven is onvoldoende.  Relevant publications of own and other research groups. Reviews alone are not sufficient. | **Most relevant publications from the project group** - Dekkers, J.C., Geenen, R., Evers, A.W.M., Kraaimaat, F.W., Bijlsma, J.W.J., & Godaert, G.L.R. (2001). Biopsychosocial mediators and moderators of stress-health relationships in patients with recently diagnosed rheumatoid arthritis. Arthritis and Rheumatism, 45, 307-316.  - Evers, A.W.M., Lu, Y., Duller, P., van der Valk, P.G.M., Kraaimaat, F. & van de Kerkhof, P.C.M. (2005). Common burden in chronic skin disease? Contributors to psychological distress in adults with psoriasis and atopic dermatitis. British Journal of Dermatology, 152, 1275-1281*.*  - Evers, AWM, Kraaimaat, FW, Geenen, R, Jacobs, JWG & Bijlsma, JWJ (2003). Stress-vulnerability factors as long-term predictors of disease activity in early rheumatoid arthritis. Journal of Psychosomatic Research, 55, 293-302.  *See also Zautra, A.J. (2003). Comments on ‘Stress-vulnerability factors as predictors of long-term disease activity in early rheumatoid arthritis’. Journal of Psychosomatic Medicine, 55, 303-304.*  - Evers, A.W.M., Kraaimaat, F.W., Geenen, R., Jacobs, J.W.G. & Bijlsma, J.W.J. (2003). Pain coping and social support as predictors of long-term functional disability and pain in early rheumatoid arthritis. Behaviour Research and Therapy, 41, 1295-1310.  - Evers, AWM, Kraaimaat, FW, van Riel, PLCM & de Jong, AJL (2002). Tailored cognitive-behavioral therapy in early rheumatoid arthritis patients at risk: A randomized, controlled trial. Pain 100,141-153.  - Evers, A.W.M., Kraaimaat, F.W., Geenen, R., Jacobs, J.W.G. & Bijlsma, J.W.J. (2002). Long-term predictors of anxiety and depressed mood in early rheumatoid arthritis: A 3 and 5-year follow-up. Journal of Rheumatology, 29, 2327-2336.  - Evers, A.W.M., Kraaimaat, F.W., van Riel, P.L.C.M. & Bijlsma, J.W.J. (2001). Cognitive, behavioral and physiological reactivity to pain as a predictor of long-term pain in rheumatoid arthritis patients. Pain, 93, 139-146  - Evers, A.W.M., Kraaimaat, F.W., van Lankveld, W., Jongen, P.J.H. & Bijlsma, J.W.J. (2001). Beyond unfavorable thinking: The Illness Cognition Questionnaire for chronic diseases. Journal of Consulting and Clinical Psychology, 69, 1026-1036.  - Evers, A.W.M., Kraaimaat, F.W., Geenen, R. & Bijlsma, J.W.J. (1998). Psychosocial predictors of functional change in recently diagnosed rheumatoid arthritis patients. Behaviour Research and Therapy, 36, 179-193.  - Evers, AWM, Kraaimaat, FW, Geenen, R & Bijlsma, JWJ (1997). Determinants of psychological distress in recently diagnosed rheumatoid arthritis. Journal of Behavioral Medicine, 20, 489-503.  - Kraaimaat, F.W., Brons, M.R., Geenen, R. & Bijlsma, J.W.J. (1995). The effect of cognitive behavior therapy in patients with rheumatoid arthritis. Behavior Research and Therapy, 53, 487-495.  - Kraaimaat, F.W. & Evers, A.W.M. (2003). Pain coping strategies in chronic pain patients: Psychometric characteristics of the Pain Coping Inventory (PCI). International Journal of Behavioral Medicine, 10, 343-363  **- Le Blanc, L.M.P., van Lieshout, A.W.T., Adema, G.J., van Riel, P.L.C.M., Verbeek M.M., Radstake TR.JD. (2006). CXCL16 is elevated in the cerebrospinal fluid in inflammatory diseases with central nervous system involvement. Neuroimmunology letters 2006 (in press).**  **- Radstake T.R.J.D, Geurts, A. Franke, B. Welsing, P.,Calandra, T., Sweep, F., Donn, R., van Riel, P.L.C.M. (2005). Rheumatoid arthritis disease severity is correlated with the genetic functional variants and circulating levels of Macrophage migration Inhibitory Factor (MIF*).* Arthritis and Rheumatism 52:3020-9.**  - Zautra, A.J., Hoffman, J., Potter, P. Matt, K.S., Yocum, D. & Castro, L. (1997). Examination of changes in interpersonal stress as a factor in disease exacerbations among women with rheumatoid arthritis. Annals of Behavioral Medicine, 19, 279-286.  - Zautra, A.J., Hoffman, J.M., Matt, K.S., Yocum, D., Potter, P.T., Castro, W.L. & Roth, S. (1998). An examination of individual differences in the relationship between interpersonal stress and disease activity among women with rheumatoid arthritis. Arthritis Care and Research, 11, 271-279.  - Zautra, A.J., Yocum D.C., Villanueava, I., Smith, B., Davis, M.C., Atrrep, J. & Irwin, M. (2004). Immune activation and depression in rheumatoid arthritis. Journal of Rheumatology, 31, 457-63.  **Other relevant publications (other than from the research group)**  - Ader, R, Felten, D.L., & Cohen, N. (2001). Psychoneuroimmunology. San Diego: Academic Press.  - Hirano, D, Nagashima, M., Ogawa, R., & Yoshino, S. (2001). Serum levels of interleukin 6 and stress related substances patients with rheumatoid arthritis. Journal of Rheumatology, 28, 490-495.  - Huyser, B*.* & Parker, J.C. (1998). Stress and rheumatoid arthritis: An integrative review. Arthritis Care and Research, 11, 135-145.  - Liew, F.Y., Wei, X.-Q., & McInnes, I.B. (2003). Role of interleukin 18 in rheumatoid arthritis. Annals of Rheumatic Diseases, 62 48-50.  - Ligier, S. & Sternberg, E.M. (2001). The neuroendocrine system and rheumatoid arthritis: Focus on the hypothalamo-pituitary-adrenal axis. In R. Ader, D.L. Felten & N. Cohen (Eds.), Psychoneuroimmunology*, Vol.2* (3ed.) (pp. 449-469). San Diego, CA: Academic Press.  - Richards, H.L., Ray, D.W., Fortune, D.G. & Griffiths, C.E.M. (2005). Response to the hypothalamic-pituitary-adrenal axis to psychological stress. British Journal of Dermatology*,* 153, 114-1120.  - Segerstrom, S.C., Kemeny, M.E., & Laudenslager, M.L. (2001). Individual differences factors in psychoneuroimmunology. In R. Ader, D.L. Felten & N. Cohen (Eds.), Psychoneuroimmunology, Vol.2 (3ed.) (pp. 87-109). San Diego, CA: Academic Press.  - Straub, R.H., Dhabar, F.S., Bijlsma, J.W.J. & Cutolo, M. (2005). How psychological stress via hormones and nerve fibers may exacerbate rheumatoid arthritis. Arthritis and Rheumatism, 52, 16-26. |

| **7** | 1. **(Global) work plan for the whole project** |
| --- | --- |
| Aangeven wat de methoden en middelen zijn waarmee de vraagstelling van het project zal worden beantwoord; globaal tijdschema vermelden.  Bij projecten met een looptijd tot 1,5 jaar hier het gedetailleerde werkplan voor het hele project formuleren  Give the methodology and means that will be used to answer the hypothesis of this project. Give global timelines.  For projects with a total running time of 1,5 year or less the detailed work plan for the complete project should be given here. | As a follow-up to the prospective study, the present project focus on an experimental approach with an experimental stress condition and a stress-management intervention. For the sake of clarity, however, all 3 parts (prospective study, experimental stress condition and stress-management intervention) will be described below:Subjects In the prospective study, 100 patients with definite RA participate. Exclusion criteria are comorbid conditions (such as psoriatic arthritis, malignancy, renal insufficiency) and psychiatric disturbances that interfere with the study protocol. Patients are required to receive stable medication and standard care for at least 3 months. Stop criteria are a change of systemic medication or standard care during study period.  For the experimental approach, a group of 64 RA patients will be randomly selected from the 100 prospective patients (32 patients with high risk and 32 patients with low risk).  Psychological risk groups are identified according to previously assessed risk factors for RA disease activity and physical complaints (Evers et al., 2001,2002a/b, 2003a/b,). Specifically, patients are classified as high risk when, compared to RA norm groups, they score in the upper 50% on at least two out of the following cognitive-behavioral factors: illness cognitions of perceived helplessness, passive avoidant coping with stress or pain and low levels of social support. Procedure   I. Prospective study during 6 months (NWO-Veni 2005-2007)  For the prospective part, clinical and laboratory assessments of disease activity and immune and neuroendocrine function as well as self-reported disease activity, physical complaints and psychological factors over the preceding 4 weeks are compiled for 100 RA patients at 7 monthly visits to the university hospital for a period of 6 months. II. Experimental study during 4 months From the patients completing the prospective study, a subgroup of 64 RA patients will be asked to participate in the stress-experiments and the stress-management intervention, including 3 visits for the experimental stress-condition at pretreatment, post-treatment and the 3-months follow-up, and (for the treatment group only) 8 visits for the stress-management intervention between pre- and post-treatment (2 weekly sessions during 4 weeks).  As for the prospective study, appointments for the assessments are made between 10.00 a.m. and 12.00 a.m., and patients are asked not to smoke, drink coffee, alcohol or exercise in the two hours prior to their appointment.  *Prospective study (6 months) Experimental study (4 months)*    7 monthly assessments points 3 assessment points with an experimental stress  during natural course condition for the treatment and control group at  week 0 (1) and week 4 (2) and week 16 (3)  1 2 3 4 5 6 7 1 2 3  Pre- Post- Follow-up  treatment treatment |
| **7** | 1. **(Global) work plan for the whole project** |
| Aangeven wat de methoden en middelen zijn waarmee de vraagstelling van het project zal worden beantwoord; globaal tijdschema vermelden.  Bij projecten met een looptijd tot 1,5 jaar hier het gedetailleerde werkplan voor het hele project formuleren  Give the methodology and means that will be used to answer the hypothesis of this project. Give global timelines.  For projects with a total running time of 1,5 year or less the detailed work plan for the complete project should be given here. | *II a. Stress-experiment*  After offering a neutral task to assess baseline data in the laboratory, the validated Trier Social Stress Task (TSST) will be applied to all 64 RA patients (Kirschbaum et al., 1993). In the TSST, patients are asked to prepare and conduct a speech for a critical audience. Previous studies have shown that particularly cortisol levels were altered during the TSST stress exposure in patients who were psychologically at risk (Fortune et al., 2005). Three equivalent TSST versions are randomly applied in patients at the different assessments points in alternated order to control for repeated measurement effects.  *II b. Stress-management intervention*  Half of the patients with a high respectively low risk profile will be randomized to the treatment or control condition. The patients in the treatment condition (n=32) receive a short-term stress-management intervention, including 2 weekly sessions during 4 weeks, consisting of validated cognitive-behavioral stress-management and relaxation techniques, with intensive daily applied relaxation exercises (Öst & Westling, 1995). This cognitive-behavioral stress-management intervention previously proved to have strong effects on stress reactivity responses in patients with affective disorders (mean ES of .80; Ost & Westling, 1995; Arntz, 2003).  The other half of the patients (n=32) is randomly assigned to the control group. These patients do not receive additional treatment during 4 months. Measurements At the assessments points of the experimental study, the same self-report, clinical and laboratory measures will be assessed as in the prospective study. Moreover, stress reactivity responses during the stress-experiment will be assessed before and after the stress exposure by self-reported distress and physical symptoms, humoral (cortisol), autonomous (heart rate variability, skin conductance) and immune responses. 1. Clinical and laboratory assessments The following measures are assessed at all assessment points during the prospective study and the experimental study.  *Disease activity* will be measured with the DAS.  *Humoral and immune measures*. Blood samples will be taken, prepared and frozen for later analyses.  Humoral response: serum assays of cortisol.  Immune response: serum analyses of IL-1, IL-6, IL-18, MIF, TNF-alpha.  During the experimental stress exposure, *autonomous response* (heart rate variability, skin conductance) are additionally assessed.  *2. Self-report assessments*  The reliability and validity of the self-report measures have been previously studied in patients with RA (Evers et al., 1998, 2001,2002, 2003). The following measures will be assessed at all assessment points during the prospective and experimental study.  *Self-reported disease activity* (RADAI) that correlates relatively highly (>.60) with clinical indicators of disease activity (DAS).  *Distress* with a SUD-scale (subjective units of distress) and the STAI (state anxiety).  *Physical symptoms* of pain and fatigue (IRGL).  *Psychological risk factors:* Illness cognitions of perceived helplessness (ICQ)*,* social support: (IRGL), passive-avoidant coping with stress (UCL) and pain (PCI)  *Stressors* of small life events, including interpersonal stressor (ISLE) and daily hassles (APL)  *Control variables* include the assessment of negative affectivity (EPQ) measured once at the first assessment point. In addition, use of medication, acute infections and health behaviors (e.g., compliance with medication, exercising, diet, alcohol use) are assessed with a questionnaire specifically developed for this study. |

| **7.** | 1. **Power calculation** |
| --- | --- |
| Geef hier de berekening en statistische en epidemiologische onderbouwing op basis waarvan het aantal patiënten, vrijwilligers of proefdieren is bepaald.  Give the power calculation and statistic and epidemiological proof, based on which the number of animals / subjects has been determined | In the present experimental study, 64 RA patients will be included (32 patients with high and 32 patients with low risk) and participate in the stress-experiments at 3 assessment points. Half of the patients with a high respectively low risk profile will be randomized to the control or the treatment condition of the stress-management condition.    For the experimental stress condition, main effects between patients at risk and not at risk are expected on subjective distress, autonomous measures (heart rate variability and skin conductance) and cortisol. Power calculations are based on measures of subjective distress (STAI) for analyses of covariance with an alpha of 0.05. Main effects with an effect size (f) of 0.35 yield a power of 0.87.  For the stress-management intervention, primary effects are expected on subjective distress between patients at risk and not at risk. Secondary outcomes include immune and humoral responses, inflammatory activity and physical symptoms. Power calculations of the stress-management intervention are based on the primary outcome measures of subjective distress (STAI) for analyses of covariance with an alpha of 0.05. Interaction effects between group (treatment vs control condition) and risk groups (high/low risk) with an adjusted effect size (f) of 0.42 yield a power of 0.90. |
| **7.** | 1. **Milestones and timelines** |
| Geef hier een onderbouwing van de geplande tijdsduur; met name ook op kritische onderdelen zoals patiëntrekrutering  Explain the expected timelines. Give special care to an explanation of critical elements like the expected rate of patient inclusion. | 3 months (01-09-2006 until 01-12-2006): Review of the literature, preparation of materials and logistics, selection of patients for the experimental approaches, preparation of data management, therapist training.  12 months (01-12-2006 until 01-09-2007): Inclusion of patients, stress experiments and stress-management intervention with pre-, post- and follow-up assessments in the first 20 patients, data management.  24 months (01-09-2007 until 01-09-2008): Inclusion of patients, stress experiments and stress-management intervention with pre-, post- and follow-up assessments in about 48 patients. Preliminary data analyses regarding the stress experiment and preparation of international publications.  36 months (01-09-2008 until 01-09-2009): Stress experiment and stress-management intervention with pre-, post- and follow-up assessments in 64 patients. Data analyses and publications of results in national and international peer-reviewed journals. |

| **7.** | 1. **Detailed work plan 1^st^ half of the project** |  |
| --- | --- | --- |
| Geef hier een gedetailleerde beschrijving van de uitvoering van het onderzoek in de periode tot de tussenrapportage (afhankelijk van de looptijd na 1, 1,5 of 2 jaar).  Please give a detailed description of the work plan for the first period of the project (until the interim evaluation at 1, 1,5 or 2 years) | Preparation of the approval of the ethical commission for the experimental part of the study: the stress-experiments and stress-management intervention.  Preparation of the inclusion procedure for patients, the logistic of the stress-experiment, particularly the application of the TSST with the psychophysiological assessments, the stress-management intervention as well as the clinical, laboratory and self-report assessments during all assessment points.  Start data collection: From a total number of 64 patients, about 36 patients are expected to participate in the stress-experiment and the stress-management intervention during the first 18 months.    Data management and preparation of statistical analyses with regard to the effects in the stress-experiment and the stress-management intervention.  Preparation of oral presentations at national and international congresses and symposia and scientific publications in internatonal peer-reviewed journals regarding   - Review about effects of psychological stresssors and risk factors in RA - Preliminary report of patients psychologically at risk vs. not at risk in the stress-experiment |  |
| **8.** | 1. **Structure and cooperation** | |
| Geef hier aan binnen welke uitgebreidere kaders/ langlopende onderzoekslijnen in uw instituut dit project ingebed is.  Geef tevens aan welke (internationale) samenwerkings-verbanden er eventueel zijn | The present proposal is part of the research program Determinants of Health and Disease of the ‘Nijmegen Centre for Evidence Based Practice’ of the Radboud University (coordination prof. dr. F.W. Kraaimaat). There is a longstanding research history concerning psychopysiological stress mechanisms in chronic inflammatory diseases, particularly rheumatic diseases (in collaboration with prof. van Riel, dr. T. Radstake), in addition to a narrow collaboration with the department of endocrinology (prof. dr. F.Sweep). This research is conducted in numerous projects in multidisciplinary settings and embedded in the UMC St Radboud  On a regional and national level, there is a narrow and longstanding collaboration with departments of rheumatology with the St. Maartenskliniek (dr. W. van Lankveld, dr. F. van den Hoogen), the Rijnstate Hospital (dr. A.J.L. de Jong) and the Jeroen Bosch Hospital (dr.P.van Ooijen). In addition, there is a longstanding co-operation with the department of Rheumatology and Clinical Immunology of the University Medical Center Utrecht (chair Prof. dr. J.W.J. Bijlsma) and the department of Health Psychology of the Utrecht University (Dr. R. Geenen). The project group also participates in national forums of social scientific research in rheumatology (SWORA and NVR). Finally, the research group participates in the national research schools ‘Experimental Psychology (EPP)’ and ‘Psychology and Health’ (P&H).  On an international level, there are several collaborations with outstanding experts in the area of rheumatology, particularly in the field of psychophysiological stress mechanisms in RA and other chronic inflammatory diseases, with prof. dr.A. Zautra (University of Arizona, US), dr. K. Thieme (University Heidelberg, G, and University of Washington, US), dr. T. Gieseke (University Michigan,US). For the present project, the collaboration with one of the main experts in psychoneuroimmunology for RA (prof. dr. A Zautra) is particularly relevant. | |
|  | 1. **Patient involvement** | |
| Geef bij patiëntgerelateerd onderzoek aan op welke wijze patiënten betrokken zijn geweest bij de vraagstelling en ontwerp van het onderzoek en of de onderzoeksvraag overeenkomt met de vragen van patiënten. | - Is this research question in line with demands and expectations of patients?   In spite of the considerable time patients spend on the study during the prospective part, all participants underline the importance and clinical relevance of the study. A substantial number of participants also spontaneously commented that they would be interested in the effects of a stress-management intervention. Consequently, the present project seems to be in line with patients’ needs. In addition, the idea that psychological stress factors only play a role in subgroups of patients at risk usually corresponds with the patients’ view that there is a high individual variability between patients regarding the effects of stress on RA. | |
|  | - Do you include input of patient’s expertise in this study? If so, how?   At this moment, patients participating in the prospective study already receive a newsletter every 6 months about the project on the theoretical background, the state of the art and the results already obtained. Comments of the participants have already resulted in several (small) logistic changes of the project. For the future, additional actions are planned, including the invitation of members of the patient organization to comment on the study protocol and the organization of patient symposia for the participants at the midpoint and end of the study. | |
|  | - In what phase of the study active involvement of patients and / or patient organizations is desirable and feasible?   Active involvement of patients and patient organizations is particularly relevant at the end of the study a) for the communication of the results to patients by newsletters, patient symposia and publications in patient journals and journals for the general public (also see point 10) and b) for the possible implementation of the study results regarding the diagnostic and treatment instruments in clinical practice (also see point 11c). | |

| **9.** | **Relevance: what is the social relevance and what is the gain for:** |
| --- | --- |
| 1. Geef hier aan waarom dit onderzoek zou moeten worden uitgevoerd. Maak duidelijk in hoeverre het onderwerp een (wetenschappelijk) onderbelicht gebied zou zijn. 2. Neem aspecten als maatschappelijke last en betekenis voor beleid (algemeen of dat van het Reumafonds) op. 3. Bij interventie-onderzoek effectiviteit en eventuele kostenbesparende factoren van de behandeling aangeven. | - Patients   It is well known that subgroups of RA patients react insufficiently to pharmacological treatments. Although patients and health professionals attribute this treatment failure partly to the effects of psychological stress factors, there is insufficient empirical evidence for psychophysiolgical stress mechanisms at present, particularly not with regard to the immunological and neuroendocrine links between stress and RA. In addition, effective psychological interventions that are known to affect the physiological stress response in RA are lacking. Research that contributes to new insights in psychophysiologcal stress mechanisms and the related therapeutic possibilities for RA patients is therefore likely to have a high priority for patients and the involved professionals. |
|  | - - Society   Insights into psychophysiological stress mechanisms and possible additional therapeutic possibilities for patients psychologically at risk poses a challenge for all professionals involved in the cure and care of patients with rheumatic diseases. A large number of RA patients are directly or indirectly associated with the patient organization of the Dutch Arthritis Association. Research that contributes to new insights in psychophysiological mechanisms can be used to further improve the diagnostic instrument for the screening of RA patients psychologically at risk. Therapeutic possibilities to change psychophysiological stress reactivity patterns of patients at risk by stress-management interventions can possibly contribute to more goal-directed and focused treatment for RA patients. Both the improved diagnostics and interventions can largely result in decreased social impact and reduced costs of RA in the future. |
|  | - - Science   The study of psychophysiological stress mechanisms contributes to our understanding of psychophysiological factors that are involved in inflammatory processes for RA as well as of possible therapeutic ways to change these psychophysiological reactivity patterns. In view of the preliminary evidence for common effects of psychophysiological stress mechanisms in various inflammatory diseases, the results are probably also relevant for other chronic inflammatory diseases.  A particular advantage of the present project is the close collaboration of different multidisciplinary experts in the field of rheumatology with regard to psychophysiology, immunology and endocrinology - a prerequisite to study the complexity of the multiple factors affecting chronic inflammatory diseases, such as RA. |

| **10.** | **Intended ways of communication to propagate the results** |
| --- | --- |
| Geef hier ook aan op welke wijze de resultaten naar **anderen dan vakgenoten** zullen worden gecommuniceerd.   1. Geef aan wat naar uw mening de **PR-mogelijkheden** van het onderzoek zijn.   Publicaties in vaktijdschriften en presentaties op congressen ed. worden voor vaststaand aangenomen. | The results of this study will be published in a number of national and international peer-reviewed journals. Furthermore, the results will be shared with other health professionals in the field of rheumatology, for example by oral presentations on the national conventions of the Dutch Association for Rheumatology (NVR) and Social Sciences in Rheumatology (SWORA) as well as international congresses. In 2006, the first results of the prospective study are already presented at different international congresses (Eular 2006, Psychology & Health 2006, International Congress of Behavioral Medicine, 2006).  Regarding other clinicians and health professionals, the screening instrument for RA patients psychologically at risk will be further adjusted and this improved version will be available to others clinicians and researchers in the field to support further implementation. When the stress-management intervention is effective, the protocol will be distributed for health professionals, researchers and therapists. Other communication strategies of presentations and publications for health professionals (rheumatologists, primary care, multi-disciplinary professionals) will be further continued (e.g. interviews: De Psycholoog 2005, publications in health professionals books: Pijninfo 2005; Psychologie&Geneeskunde, 2006).  Finally, using the broad contacts of the research group with patient organisations (including RPB), various activities will be performed in the future to make the results available to patient organisations by oral presentations, newspapers and web publications. Information about this study has already been presented at different patient symposia and published in journals for the general public (e.g., Plus Magazijn, 2005, Leef, 2005). In addition, the participants already receive regularly newsletters about the content of the study, theoretical backgrounds and the results. |
| **11.** | **Objects at the end of the study** |
|  | 1. **Expected ‘product’ at the end of the study** |
| Wat is het tastbare resultaat van dit onderzoek: kan hier een in de praktijk bruikbare interventie uitkomen, geeft de uitkomst een mogelijkheid tot preventie, wordt een nieuw diagnostisch of prognostisch hulpmiddel onderzocht?  Bij fundamenteel onderzoek aangeven wat de verwachte volgende stap zal zijn. | Clinical practice:  - Regarding the diagnostic instrument to screen for patients psychologically at risk, the present study contributes to a further validation of the instrument that have been developed in previous research of the project group and that is currently used in rheumatology practice. With the present project, the instrument will be further validated, particularly with regard to the interaction with shorter and longer-term stressors and specific physiological correlates.  - In the case that the stress-management intervention is effective in changing the stress-response of RA patients psychologically at risk, a possible further therapeutic tool for tailored interventions for these patient groups becomes available for clinical practice. The treatment protocols will then become available at the end of the project for researchers as well as therapists in rheumatology practice.  Publications / presentations / symposia:  - Publications in the main scientific peer-reviewed journals, international as well as national  - Publications for patient organizations and health professionals  - Oral presentations at international and national conferences and symposia for researchers, health professionals and patient organizations |

|  | 1. **Intended follow-up research** |
| --- | --- |
| Geef aan wat bevorderende en belemmerende factoren kunnen zijn bij vervolgonderzoek  Denk hierbij bv. aan financiële belemmeringen en draagvlak voor de toepassing. Geef aan of u of anderen al tijdens het onderzoek stappen kunnen ondernemen om op financiële belemmeringen te anticiperen of om het draagvlak te vergroten. | Generally, there are two main research strategies for follow-up projects, focusing on fundamental questions about psychophysiological stress mechanisms for patients at risk and the more patient-oriented research about diagnostic and therapeutic possibilities. For the fundamental part, research will be directed at the specification of the stress-disease relationships and the specific immunological and neuroendocrine mediators that form a possible link between stress factors and RA for patients at risk.  When the present distinction in patients risk profiles is further supported by immune and neuroendocrine factors and the stress-management intervention is effective for patients at risk, efforts will be also directed to the further implementation of the screening- and treatment-methods in other rheumatology clinics. These implementation efforts are facilitated by the broad set of preparation strategies that are already realized during the study period (see also point 10, 11a en 11c). In addition, future studies can particularly focus on the further improvement of the diagnostic screening and treatment methods, for example, identifying the essential working elements in the stress-management intervention for patients at risk.  In view of the preliminary evidence for common effects of psychophysiological stress mechanisms in different chronic diseases, the results may be also relevant for other chronic inflammatory diseases. Follow-up research on the effects of psychophysiological stress mechanisms in other chronic inflammatory disease in comparison to RA and the study of the disease specific and generic effects are also of particular relevance. |
|  | 1. **Implementation of study result/ Possibility of ‘technology transfer’** |
| De mogelijkheden tot implementatie/ technology transfer aangeven per doelgroep: professionals, deelnemers aan het onderzoek en de patiëntengroep.  Bij toegepast onderzoek aangeven of de verwachtte resultaten direct in de praktijk toepasbaar zijn, zo niet aangeven welke vervolgstappen nog nodig zijn.  Bij fundamenteel onderzoek in ieder geval aangeven hoe de resultaten van het project gepresenteerd worden aan vakgenoten en welke middelen hiervoor gebruikt kunnen worden, naast publicaties in vaktijdschriften. | For clinical practice, additional education for health professionals about psychophysiological stress mechanisms and further implementation of the screening instruments and treatments for patients at risk in clinical practice can be expected:  - Regarding the diagnostic instrument to screen for patients psychologically at risk, the present study contributes to a further validation of the instrument that has been developed in previous research of the project group and that is currently regularly used in rheumatology practice. Further systematic implementation strategies in other rheumatology clinics are under preparation.  - In case the stress-management intervention is effective, a possible further therapeutic tool for tailored interventions for these patient groups becomes available for clinical practice. At this moment, tailored psychological interventions for RA patients at risk are already part of regular rheumatology practice. The treatment protocols will become available at the end of the project for researchers as well as therapists in rheumatology practice and further systematic implementation strategies are currently under preparation.  - The knowledge about psychophysiological stress mechanisms in RA patients will be integrated in the education of rheumatologists and other health professionals in the area of rheumatology through the participation of the study group in educational programs for rheumatologists and health allied professionals, oral presentations and workshops at symposia and conferences as well as publications in national and international study books and journals (see also point 10). |

| **12** | Personnel | | | | |
| --- | --- | --- | --- | --- | --- |
|  | 1. Participants (not to be financed) | Title | Specialty | Institute (employer) | hour/week |
|  | A.W.M. Evers  F.W. Kraaimaat  P.L.C.M. van Riel  **T.R.J.D** Radstake  F.C. Sweep  A.J. Zautra | Dr.  Prof.  Prof.  Dr.  Prof.  Prof. | Psychologist  Psychologist  Rheumatologist  Rheumatologist  Endocrinologist  Psychologist | UMC St Radboud  UMC St Radboud  UMC St Radboud  UMC St Radboud  UMC St Radboud  Arizona State University (US) | 2  1  1  1  1  1 |
|  | 1. Participants to be financed  (in total max. 1 FTE) | Title | Specialty | Institute (employer) | hour/week |
|  | Junior researcher  Therapist | Drs.  Drs. | Psychologist  Psychotherapist | UMC St Radboud  UMC St Radboud | 30  6 |

| **13** | Financial information | | | |
| --- | --- | --- | --- | --- |
|  | Contact at financial dept. | Name: | Dhr. J. Wijnia | |
|  |  | Address: | UMC St Radboud  840 Medische Psychologie  Postbus 9101  6500 HB Nijmegen | |
|  |  | Place: | Nijmegen | |
|  |  | Telephone: | 024-3613608 | |
|  |  | Fax: | 024-3613425 | |
|  |  | E-mail: | j.wijnia@mps.umcn.nl | |
|  | | | | |
| **14** | **Co-financing requested elsewhere:** | | | Decision expected on: |
|  | NWO-Veni and UMC St Radboud for the prospective part  of the study. | | | Already granted until end 2007 |

| **16** | **Motivation of requested finance for:** |
| --- | --- |
| Altijd invullen  Always complete this section | - Personnel   A junior researcher (30 hrs per week) is requested for the inclusion of patients, the development and application of the stress-experiment, the coordination of all assessments at pre- and post-treatment and the follow-up of the stress-management intervention, the psychological test assessments, data analyses and preparation of the publications. A psychotherapist (6 hrs per week) is requested to perform the stress-management interventions. |
|  | - Materials   Material costs primarily include clinical and laboratory assessments of disease activity and immune and neuroendocrine measurements, in addition to the costs of the autonomouse measures during the stress-experiment. In addition, repeated assessments of psychological tests are required (including posting costs). |
|  | - Other costs and apparatus and other investments   Other costs include patient travel expenses. |
